# Tackling financial insecurity for autoimmune rheumatic diseases in developing countries in sub-Saharan Africa is of utmost importance

**DOI:** 10.11604/pamj.2024.47.81.42698

**Published:** 2024-02-22

**Authors:** Jan René Nkeck, Adeline Pelda, Madeleine Ngandeu-Singwé

**Affiliations:** 1 Cameroon College of Rheumatology, Yaoundé, Cameroon,; 2 Yaoundé Rheumatology Research Team, Yaoundé, Cameroon,; 3 Department of Internal Medicine and Specialties, Faculty of Medicine and Biomedical Sciences, University of Yaoundé I, Yaoundé, Cameroon

**Keywords:** Financial insecurity, autoimmune rheumatic diseases, sub-Saharan Africa

## To the editors of the Pan African Medical Journal

Autoimmune rheumatic diseases (AIRDs) are still considered rare in sub-Saharan Africa (SSA). They are dominated by rheumatoid arthritis, systemic lupus, other connective tissue diseases (systemic sclerosis, primary Sjögren's syndrome, idiopathic inflammatory myopathies, etc.), and systemic autoimmune vasculitis [1]. These conditions are associated with high mortality rates due to systemic damage, dominated by renal, cardiovascular, and respiratory complications; on the other hand, morbidity is impressive, affecting all organs, in particular the skin, joints, muscles, and the nervous system, exposing sufferers to serious physical and/or psychological disability. Diagnosing AIRDs requires costly tests that are not always accessible. Their management is multi-disciplinary, necessitating frequent consultations with different specialists, and multiple costly long-term treatments, most of which, in particular biologics, are not easily accessible nor available in developing countries [2]. For developed countries, the costs associated with AIRDs have driven up healthcare costs, with annual estimates topping $100 billion [3]. The direct annual cost of treating rheumatoid arthritis, for example, is $10.9 billion, while indirect costs alone total $8.4 billion in the United States of America [3]. For developing countries, the burden of care for these conditions is immense, and falls almost entirely on the shoulders of patients and their families, who are often in a vulnerable financial situation, and few covered by health insurance [2]. To improve the state of AIRDs care in developing countries in SSA, it is essential to effectively address the financial insecurity of patients, in order to improve health policies and the optimal use of resources, especially in our context in which the latter are scarce.

### Understand the financial burden of autoimmune rheumatic diseases

**Direct costs:** these represent the share of expenditure directly linked to healthcare, and are generally dominated by hospitalization, the acquisition of medication, complementary tests, and treatment follow-up [4]. There is a clear relationship between financial income and chronic disease. People with low incomes are more likely to have difficulty adhering to health behavior change recommendations and receiving recommended surveillance tests. Financial precarity will lead to a delay in consultation, and a delay in management, and will inevitably be linked to a poorer prognosis. Socioeconomic status, including income and occupation, is inversely related to morbidity and mortality, and people who are socially and economically well-off are likely to enjoy better health because they are more likely to consult a doctor [4]. The treatment of autoimmune diseases has improved markedly with the advent of biologics. Although these molecules offer broader alternatives to patients who do not respond optimally to conventional drugs, they entail an increased financial burden, with annual costs ranging from $25,000 to $40,000 in the case of rheumatoid arthritis for example [5].

**Indirect costs:** this is the share of expenditure incurred by individuals and society outside the healthcare system. It takes into account transportation to care centers, the purchase of over-the-counter medication, patient nutrition, and household support. Societal cost refers to factors involving work absence, reduced productivity, and disability-related inability to work [5]. AIRDs impose an enormous burden on society in terms of both morbidity and mortality. They entail a significant social and economic burden, affecting individuals' quality of life and ability to work. Education, employment status, income, family, and social stress all play a part. Social problems develop as chronic symptoms worsen, making mobility difficult, leading to self-care difficulties and social isolation [5]. Nearly 50% of patients will have impaired productivity, while 25% will be recognized as disabled workers. The rising rate of AIRDs is a major contributor to increased medical expenditure, making it difficult to provide financial access to care and to extend health coverage to all people. These conditions also affect the quality of the work-force, such as productivity, reduced wages, and earnings, and lead to early retirement, staff turnover, and disability [6,7].

**Intangible cost:** AIRDs does not spare patients' sex lives, as persistent joint pain, stiffness, and extreme fatigue can alter the frequency of sexual intercourse - due to the difficulty of adopting a comfortable position during intercourse - and also impair libido as body image esteem declines [8]. These various problems may be responsible for a delay in conception. What's more, the side-effects of medication alone can be a source of reduced fertility, especially in men [9]. AIRS alters the quality of life of patients' children, making it difficult for them to attend school and increasing the financial outlay required for their care. The daily lives of these patients will also be affected, as the most basic activities such as travel, housework, sports, toileting, leisure, family visits, outings, and others will be difficult to carry out [10].

**Prospects for reducing financial insecurity:** on an individual level, we need to promote the social integration of patients through associations and group therapies, as part of multidisciplinary care. Learning about the experience of living with the disease, having the right information to understand it, and being able to share your pain with other people in the same situation on a daily basis can be a great comfort. A socio-economic assessment is also needed at individual level to identify people in financial insecurity. At the community level, improving the quality of life of people suffering from AIRDs requires the community to be fully informed about what the disease is, its treatment, and the need for psychosocial and financial support. At a national level, it's important to work towards health insurance to cover AIRDs, to set up specialized and subsidized care centers, to improve the availability and cost of therapeutics for AIRDs, to develop policies for the social reintegration of patients, and to encourage structures to employ people suffering from these diseases.

**Conclusion:** AIRDs have a heavy financial burden in developing countries. The indirect cost of the disease, which is difficult to measure, is as high as, if not higher than, the direct cost of the disease. These diseases cause considerable damage to patients, their families, and their professional activities. It is therefore urgent to address this financial insecurity in this specific population, in order to contribute effectively to the management of these conditions.
